# ‘Safety by DEFAULT’: introduction and impact of a paediatric ward round checklist

**DOI:** 10.1186/cc13055

**Published:** 2013-10-11

**Authors:** Sanjiv Sharma, Mark J Peters

**Affiliations:** 1 Paediatric Intensive Care Unit, Great Ormond Street Hospital NHS Foundation Trust, Great Ormond Street, London WC1N 3JH, UK; 2 Critical Care Group, Portex Unit, Institute of Child Health, University College London, 30 Guilford Street, London WC1N 1EH, UK

## Abstract

**Introduction:**

Poor communication is a source of risk. This can be particularly significant in areas of high clinical acuity such as intensive care. Ward rounds are points where large amounts of information must be communicated in a time-limited environment with many competing interests. This has the potential to reduce effective communication and risk patient safety. Checklists have been used in many industries to improve communication and mitigate risk. We describe the introduction of a ward round safety checklist ‘DEFAULT’ on a paediatric intensive care unit.

**Methods:**

A non-blinded, pre- and post-intervention observational study was undertaken in a 12-bedded Level 3 tertiary PICU between July 2009 and December 2011.

**Results:**

Ward round stakeholders subjectively liked the checklist and felt it improved communication. Introduction of the ward round checklist was associated with an increase in median days between accidental extubations from 14 (range 2 to 86) to 150 (56 to 365) (Mann–Whitney *P* <0.0001). The ward round checklist was also associated with an increase in the proportion of invasively ventilated patients with target tidal volumes of <8 ml/kg, which increased from 35 of 71 patients at 08.00 representing a proportion of 0.49 (95% CI 0.38 to 0.60) to 23 of 38 (0.61, 0.45 to 0.74). This represented a trend towards an increased proportion of cases in the target range (z = 1.68, *P* = 0.09).

**Conclusions:**

The introduction of a ward round safety checklist was associated with improved communication and patient safety.

## Introduction

Communication failures are very common causes of errors and harm in medicine [1]. Intensive care units (ICUs) are one of several clinical environments that characteristically have high numbers of staff, time pressures and patients with complex and often rapidly changing needs. As a result there are typically multiple demands on any individual’s attention arising from monitors, patients, other ICU staff and visiting teams. This environment is unforgiving of poor communication [2].

The aviation industry recognised the utility of checklists as defence against the limitation in human memory and attention as far back as 1930 [3]. Clinical checklists improve patient safety, especially in complex environments with multiple potential distractions. The World Health Organisation (WHO) Surgical Safety Checklists reduce death and surgical complications in very diverse healthcare setting around the world [4]–[8]. Paediatric surgical safety improves with the use of the WHO checklist [9].

Ward round checklists are not in widespread use. One ‘FAST HUG’ checklist has been described for use in adult intensive care. This checklist was designed for the individual bedside nurse to confirm adherence to evidence-based practice rather than as a team exercise [10]. Improvements in ventilator-associated pneumonia rates have been reported following adoption of ‘FAST HUG’ [11].

One study provides support for the use of a ward round checklist on an adult ICU with increased attention to the issues that the team felt needed to be discussed for every patient every day [12]. Team building and collegiality were also increased as a result of the multidisciplinary team using an evidence-based tool to optimise patient care.

Within our unit, we identified ward round bedside communication as specific risk. We felt that ward rounds should be the key decision-making events with the whole team contributing to, and agreeing, a plan for the next 12 hours. We were concerned that information may be being miscommunicated, misunderstood or simply not heard. Any of these failings would reduce ‘situation awareness’ and reduce consistency of the mental model of the patients that are communicated across handovers to ensure delivery of high levels of longitudinal care [13]. Specific local factors that may have contributed to communication difficulties included the number of staff on ward rounds (often more than 20 people). In response to this, we adapted the principle of a ward round checklist and developed our own acronym, which aimed to confirm agreement between the team on key elements of paediatric intensive care management. We describe the impact of adoption of a ward round safety checklist to a paediatric intensive care setting.

### Aim

Our aim was to improve the effectiveness of information sharing on ward rounds in our paediatric intensive care unit (PICU).

## Methods

A prospective, non-blinded, pre- and post-intervention observational study was undertaken in a 12-bedded Level 3 tertiary PICU between July 2009 and December 2011.

The study was discussed with the Bloomsbury (London) Research Ethics Committee who confirmed that the need for ethical approval was waived.

### Planning

The risk action group designed a mnemonic aimed to cover important aspects of patients’ care that were necessary to discuss at each bedside interaction.

In preparation for introduction of the checklist, all stakeholders in the process including consultant intensivists and senior nurses were invited to comment or amend the proposal.

The DEFAULT mnemonic was developed for use to ensure that the following were discussed: D-Do Not Attempt Resuscitation status, E-Endotracheal tube fixation reviewed as secure and cuff inflated appropriately, F-Fluid management - agreement of type, allowance and overall balance target within next shift, A-Analgesia (and sedation) plan agreed and weaned if possible, U-Ulcer prophylaxis (gastrointestinal and skin), L-Lines (venous and arterial) sufficient or to be removed if possible and T-Tidal volume less than 8 ml/kg (Table 1).

**Table 1:** The DEFAULT mnemonic

**D**	**DNR status clear?**
	*‘Jimmy is for full resuscitation’*
**E**	**Endotracheal tube and cuff is safe**
	*‘Jimmy’s ETT was retaped yesterday, is secure, the cuff is inflated and the cuff pressures are in range’*
**F**	**Fluid strategy/Feeding plan agreed**
	*‘Jimmy is on 80 mls/kg, to commence feeding, if tolerated, increase to 100 ml/kg. On frusemide infusion aim of 200 mls negative balance by morning’*
**A**	**Analgesia/sedation**
	*‘Jimmy is on morphine and midazolam infusions, to commence enteral sedation and reduce to 20 mcg morphine and 2 mcg midazolam’*
**U**	**Ulcer skin and gut**
	*‘Jimmy is on ranitidine, and nursed on an airflow bed, however, his skin is fragile but not broken’*
**L**	**Lines out**
	*‘Jimmy has one radial arterial, three peripheral and a right femoral line. The femoral line is to be removed today’*
**T**	**Tidal volumes <8 ml/kg**
	*‘Jimmy has tidal volumes of 6 ml/kg’. Or ‘Jimmy is oscillated, tidal volumes not measured’*

### Implementation

As implementation was to be led by the consultants and senior nursing staff, agreement from these groups in particular and commitment to a daily change in practice was necessary. Subsequent to this consultation phase, strategies for dissemination of information regarding the checklist to junior doctors and the nursing team have included discussion at the unit meeting, emails and printed stickers to act as visual prompts.

From 1 July 2009 the ward round checklist was introduced on morning rounds. Continuity on daily rounds from a senior fellow ensured rigorous adherence to the change in practice and reinforced the role of the checklist.

After each bedside discussion of the patient the team ran through the ‘DEFAULT’ checklist. Initially this was led by the duty consultant intensivist. This was done aloud, to all members of the round and issues that had been missed or were not clear to all members of the team were discussed as prompted by the checklist.

### Assessment

Six outcome measures were assessed:

1) The proportion of ward round bedspace visits in which the DEFAULT checklist was observed to be performed.

2) The number of ‘issues’ identified during the checklist (defined as a need for further discussion or clarification by another member of staff, for example adjustment or confirmation of the prescribed fluid volumes).

3) The length of time (seconds) between the start of the DEFAULT checklist until its completion.

Measures 1 to 3 were assessed on 10 consecutive weekdays after implementation.

4) Qualitative staff satisfaction and comments. Staff satisfaction was assessed by an ‘anonymised’ questionnaire provided to all medical and nursing staff. A total of 24 questionnaires were distributed to all nursing and junior medical staff members on a day shift two months post the introduction of the DEFAULT mnemonic.

5) The frequency of episodes of accidental extubation. Because of the relatively low incidence of these events, comparison of accidental extubations rates is made between the one-year period prior to implementation of DEFAULT and two years afterward.

6) The proportion of patients with inspiratory tidal volumes estimated on the Servo-i or or Evita-4 ventilators <8 mls/kg. Inspiratory tidal volume is recorded on all patients at least at hourly intervals on computerised records (CareVue, Phillips, Eindhoven, Netherlands). Cases less than 10 kg body weight were excluded from this analyses as the potential error in measured tidal volume was considered to be too great [14] as were those not receiving controlled breaths (predominantly continuous positive airways pressure +/− pressure support). No adjustment was made for endotracheal tube leak as practice was not altered in this regard during the period of the study.

Tidal volume data was collected for the 8.00 (pre-ward round) and 12.00 (post-ward round) for one month prior to, and again immediately after, the introduction of DEFAULT. Required changes would be made by medical or bedside nursing staff. This analysis was repeated at month 6 and month 9 after implementation.

The first four parameters are potentially vulnerable to bias and hence we planned to assess the impact of DEFAULT prospectively from 1 September 2009 (two months after implementation) to reduce the risk of describing a honeymoon effect or of a variable degree of staff experience of the intervention in the early phase. Accidental extubation rates and inspired tidal volumes were routinely collected on the unit thought to be less prone to bias. Hence impact of DEFAULT was assessed immediately by reviewing changes in these routine data.

#### Statistics

Non-parametric data are presented as medians and interquartile ranges and groups are compared with the Mann–Whitney test. Categorical data are presented as proportions with 95% confidence intervals of proportion and compared with the significance of the difference between two independent proportions (z-ratio). Statistical analyses were performed on SPSS 20 for Mac (IBM SPSS, Chicago, IL, USA).

## Results

### Implementation

Very early during the initial pilot period of two months it was decided the checklist should be led by the bedside nurse rather than the consultant conducting the round. This gave the bedside nurse a platform for communication and empowered him/her to lead at least one element of the communication episode.

### Completeness, duration and ‘number of issues’

When audited over two weeks at the end of the two-month introduction period, 100%, of 103 (95% confidence interval (CI) 96 to 100%) observed ward round bed space interactions employed DEFAULT, the median time for completion was 27 (interquartile range (IQR) 12 to 105) seconds and there were a median of 1.4 (IQR 0 to 5) ‘issues’ per bed. Subsequent re-audits at 12 and 24 months have confirmed 100% use of DEFAULT on ward rounds.

### Subjective feedback

Of the 24 questionnaires distributed, 12 were returned. Subjectively, feedback from nurses was that they ‘loved it’ as it improved the feeling of involvement in the ward round process and provided a platform to air issues. Comments included that the mnemonic helped improve memory recall and allowed for main or key points of care to be addressed on the ward round, facilitating effective planning. They reported that the DEFAULT checklist indirectly gave structure to staff for discussion of key points of care helped provide guidance particularly for more junior bedside nurses. Nursing staff subsequently developed their own *aides-memoires* and the DEFAULT acronym is now printed at the top of their daily fluid charts and onto the back of their clipboards. This was mostly for the benefit of newer or junior staff who may forget what the letters stand for. A summary of comments collected from the multidisciplinary team two months after the introduction of default is shown in Table 2.

**Table 2:** Feedback from multidisciplinary team on use of DEFAULT mnemonic two months post introduction

**Positive comments**	• Love it
	• Helps to ensure vital information is not missed
	• Helps nurses who might not have done the respiratory module to check the ventilation more closely
	• Good systems overview
	• Helps reiterate many plans from ward round
	• Works well
	• Mnemonic helps easier learning and improves the memory or recall
	• Allows for main/key points of care to be addressed on ward round and effective planning
	• Gives structure indirectly to staff on discussion or key points and guidance for bedside nurse on ward round
	• Addresses the DNAR (Do Not Attempt Resuscitation) status formally on a daily basis
**Negative comments**	• Not comprehensive enough
	• Not always adhered to
**Areas for suggested development**	• Could be a more generic mnemonic so all valuable information is included for all types of patient, that is head, respiratory, orthopedic
	• Should include ‘S’ for social issues

The doctors felt it was a useful tool that usually brought up something to be discussed further. They also appreciated that asking the bedside nurse to ‘DEFAULT’ facilitated in providing structure to the ward round and helped ‘frame’ each patient interaction.

### Accidental extubation

**Figure 1:**
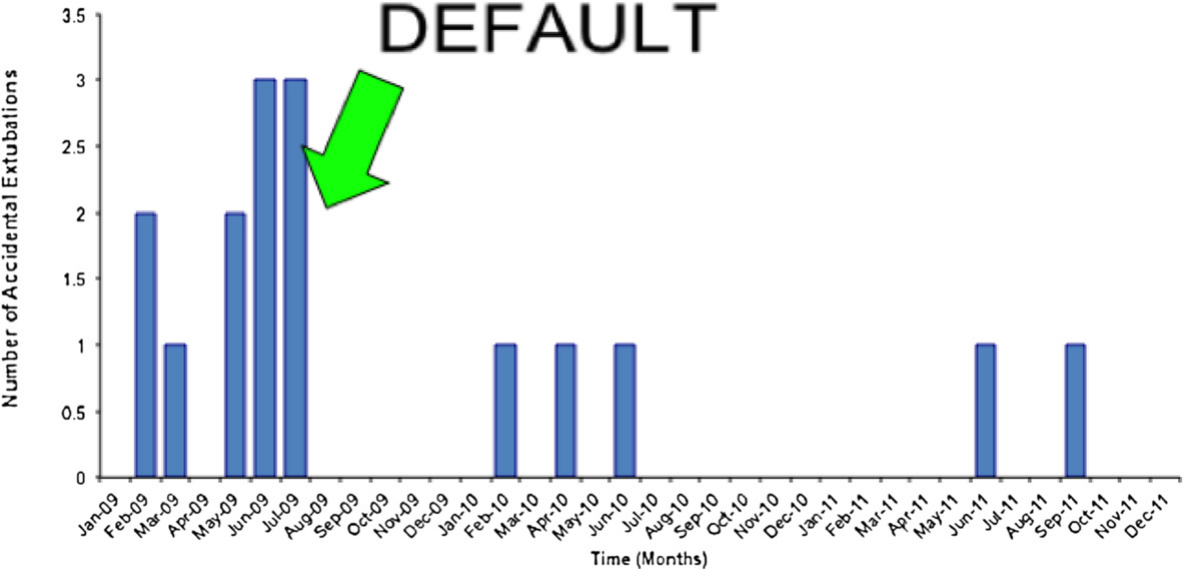
Episodes of accidental extubation by month.

**Figure 2:**
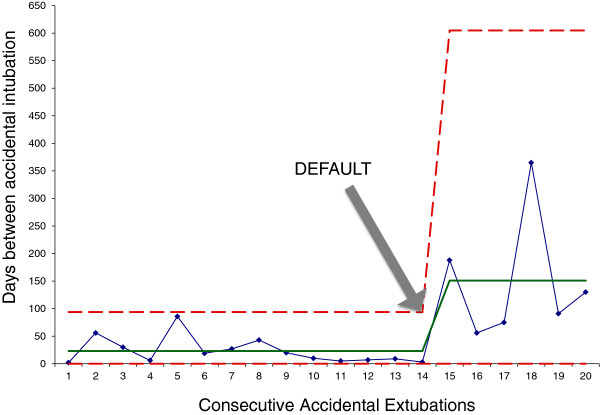
**G-chart of interval between accidental extubations.** Median (solid line) and upper and lower control limits (dashed lines) are shown. Fourteen accidental extubations were recorded in prior to DEFAULT and six afterwards. The time between accidental extubation events significantly increased following DEFAULT: median pre: 14 (range 2 to 86) days, post: 150 (56 to 365) days (Mann–Whitney, *P* <0.0001).

There was a sustained fall in the accidental extubation rate between early 2009 and the end of December 2011 (Figure 1). Overall, the number of days between accidental events increased from a median of 14 (range 2 to 86) days prior to DEFAULT to and 150 (56 to 365) days post-DEFAULT (Mann–Whitney, *P* <0.0001) (Figure 2).

### Estimates of tidal volume

Prior to the introduction of DEFAULT 35 of 71 invasively ventilated patients meeting inclusion criteria during a calendar month on PICU had a tidal volume <8 ml/kg at 08.00; this represents a proportion of 0.49 (95% CI 0.38 to 0.60). This was not significantly different at 12.00, when 29 of 67 patients had a tidal volume <8 ml/kg, (0.43, 0.32 to 0.55). Following the introduction of DEFAULT, 23/38 (0.61, 0.45 to 0.74) invasively ventilated patients, had tidal volumes <8 ml/kg at 8.00, and 25/43 patients at 12.00, (0.58, 0.43 to 0.72). This represented a trend towards an increased proportion of cases in the target range (z = 1.68, *P* = 0.09).

**Figure 3:**
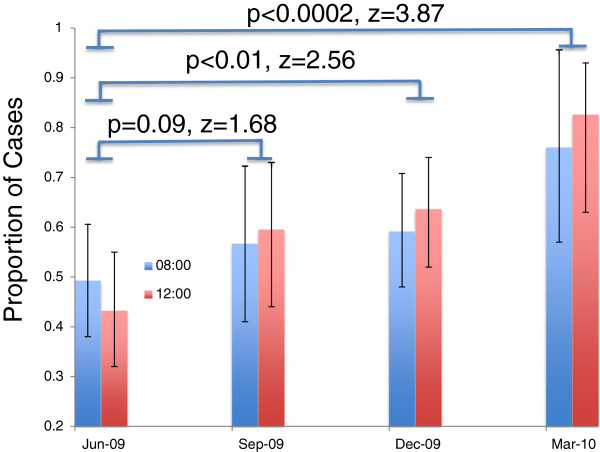
**Proportion of ventilated cases with inspired tidal volume <8 mls/Kg body weight pre- and post morning ward round.** Proportions and 95% confidence intervals of proportions are shown. Probabilities derived from two-tailed test of difference in proportions.

At six and nine months after DEFAULT the proportion of cases in the target range were significantly greater than baseline (Figure 3) (six months: at 8.00, 21/37, (0.56, 0.41 to 0.71) at 12.00, 49/77, (0.63, 0.52 to 0.74) (*P* <0.01) and at nine months at 8.00: 19/25, (0.76, 0.57 to 0.89), at 12.00, 18/22 (0.82, 0.68 to 0.91) (*P* <0.0002).

Interestingly, the increased proportions with tidal volumes <8 mls/kg remained equivalent at 8.00 and 12.00.

## Discussion

Good communication within teams is essential for good clinical care. Handovers have been identified as danger points where information needs to be passed from one team to another in a complete and succinct way. As working practices have changed ward rounds often become tools for night teams to pass information to day teams, for clinical discussions to be held and management plans of care for each patient to be made. Historically, these rounds have been led by clinicians with little participation from the wider multidisciplinary team. In contrast, the rounds within our unit are large with many non-clinical staff involved. This environment can be intimidating for more junior medical staff handing over from the previous night and daunting for nursing staff who have felt unable to contribute to discussions despite being aware of the details of clinical progress for their patient over the preceding hours and having the responsibility for carrying out the clinical plans for the shift.

Checklists within medicine have been used in a formalised way within the operating theatre to avoid harm. Recent reports have described checklists within an intensive care ward round setting but to date there has been no description of a checklist used as a team exercise in a paediatric setting. Our aim was to ensure that we were comprehensive in addressing key aspects of patient care. We looked to facilitate a change in culture whereby after each patient interaction the clinical management plan was reiterated in a structured way. By using a checklist tool our hope was to improve bedside communication, improve teamwork and improve the feeling of involvement by key stakeholders. Key aims were that the bedside nurse would be expected to reflect back the agreed care plan to the ward round group and that they should feel empowered to contribute to and influence the management plans. In this process we aimed to challenge any perceived authority gradient or hierarchy of communication.

The feedback from multidisciplinary staff has been overwhelmingly positive. Nursing staff in particular have reported the sense of empowerment and the expectation of them reflecting back the plan for the shift within the framework of a checklist has provided a platform for them to speak up and contribute to clinical conversations. Medical staff of all levels have reported that using the DEFAULT checklist has been useful as a communication tool to ensure that all details of care are discussed with a plan for the day and that it clearly punctuates the end of a patient discussion to the whole of the multidisciplinary team. In this way it appears to have facilitated the ‘flow’ of the round rather than extending its duration.

There are a number of limitations of this study. We acknowledge observer bias inherent to ‘before-and-after’ studies. We have attempted to minimise this risk by focusing on areas in which data collection was routine (tidal volume and accidental extubation) and automated to some degree. A further flaw is that several components of acronym have not been assessed. Either because some parameters on the DEFAULT list represent very rare events (for example D, lack of clarity about resuscitation status), or those for which no gold standard care is defined (for example F-fluid balance strategy or A-analgesia/sedation plan). We could not devise an effective way of assessing these parameters not least because many of our patients leave intensive care and are repatriated to their local hospitals or host wards and are under the care of different teams with limited intensive care follow-up. While it is tempting to try to assess the numbers of days of patients spend with invasive lines (L-lines out) and to assess if this might have fallen following DEFAULT, defining when a line was or was not appropriate for a case-mix adjusted population was outside the scope of this work. Comparing central line-associated infection rates and ventilator-associated pneumonia rates before and after use of the ward round checklists was considered. However, rates for these are low on our unit after the introduction of specific care bundles so as make this comparison uninformative. Intensive care bed days are not comparable due to the multiple confounding variables such as seasonal variability in emergency referral. Therefore statistical proof of efficacy is challenging. Hence we have used what data are available but also considered subjective experiences of the altered processes.

The strongest suggestion of DEFAULT improving safety comes from the comparison of accidental extubations before and after introduction of the checklist. While there was no other change in practice with respect to tube fixation on the intensive care unit during this period, accidental extubation rates are now (from 1 April 2011) reported to UK National Health Service commissioners. In theory, foreknowledge of this change may have altered in reporting practice. Regardless of the cause, we observed a clear reduction in the number of accidental extubations.

Prior to introduction of DEFAULT there was no difference in the proportion of children ventilated ≤8 ml/kg between the 8.00 or pre-ward round tidal volumes and the 12.00 or post-ward round tidal volumes. On comparison of tidal volumes at these times after the DEFAULT checklist introduction period, there was no increase in the proportion of ventilated patients with tidal volumes in the target range. Three months later, there was an increase in the proportion of patients with tidal volumes in the target range after ward round use of DEFAULT. More importantly, there was an improvement in the overall proportion of patients who were ventilated with tidal volumes in the target range before and after the ward round. This suggests that ‘DEFAULT’ influenced daily thought processes and such that safe target ranges for ventilation are an overt goal of our care for all staff.

We believe that ownership of a locally devised checklist has facilitated its introduction into this particular area and has ensured a change in culture in bedside communication. All members of the multidisciplinary team had been consulted prior to introduction of the DEFAULT checklist and were invested in its ongoing use. The development of *aides-memoires* by the nursing team for the nursing team has exemplified the commitment to continue use of this checklist at every patient’s bedside.

With time, the authors have also become aware of the danger of the checklist being recited as a mantra, such that each part of the checklist is recited unconsciously. This has the consequence that there is little thought for the importance or significance of each part of the checklist and a rehearsed DEFAULT is communicated without incorporating the ward round decisions. This has been particularly noticeable in the younger members of staff who have focused on DEFAULT as the point at which they are expected to contribute in the ward round process. The authors hope that vigilance to this and education will help overcome this.

## Conclusions

Introduction of a ward round checklist, the acronym DEFAULT, has been popular with all members of the multidisciplinary team. It is well liked by the nursing team who has seen it provide a platform for them to speak and contribute to discussions in the large multidisciplinary ward rounds, overcoming perceived hierarchies of communication. The DEFAULT checklist has facilitated effective communication thereby reducing the risk of lost information at danger points during discussions regarding patient care. Its introduction has been associated with an increase in the proportion of paediatric patients ventilated with tidal volumes in the target range and a fall in the accidental extubation rate. In a paediatric intensive care setting use of a ward round checklist used systematically for every patient can reduce risk and improve patient care.

## Key messages

● Effective communication during large multidisciplinary ward rounds is both crucial and difficult

● A simple spoken checklist may improve ward round communication

● Measures of patient safety including accidental extubation rates may be improved by simple structured communications about the control measures

● The effectiveness of a checklist is likely to depend on active, unrehearsed participation by the team

## Abbreviations

NICU: Neonatal intensive care unit; PICU: Paediatric intensive care unit; WHO: World Health Organization.

## Competing interests

The authors declare that they have no competing interests.

## Authors’ contributions

MP conceived of the study and designed the first draft of the checklist. This was revised with the RAG (see below). MP and SS planned the study and intervention. RAG members ensured implementation of the checklist. The SS and MP collected the data, MP analysed the data and both SS and MP prepared the manuscript. All authors read and approved the final manuscript.
